# Extracellular Vesicles in Pathophysiology: A Prudent Target That Requires Careful Consideration

**DOI:** 10.3390/cells13090754

**Published:** 2024-04-26

**Authors:** Sanjay Shahi, Taeyoung Kang, Pamali Fonseka

**Affiliations:** Department of Biochemistry and Chemistry, La Trobe Institute for Molecular Science, La Trobe University, Melbourne, VIC 3086, Australia; s.shahi@latrobe.edu.au (S.S.); t.kang@latrobe.edu.au (T.K.)

**Keywords:** extracellular vesicles, exosomes, pathophysiology, normal physiology, delivery vehicles, therapeutic target

## Abstract

Extracellular vesicles (EVs) are membrane-bound particles released by cells to perform multitudes of biological functions. Owing to their significant implications in diseases, the pathophysiological role of EVs continues to be extensively studied, leading research to neglect the need to explore their role in normal physiology. Despite this, many identified physiological functions of EVs, including, but not limited to, tissue repair, early development and aging, are attributed to their modulatory role in various signaling pathways via intercellular communication. EVs are widely perceived as a potential therapeutic strategy for better prognosis, primarily through utilization as a mode of delivery vehicle. Moreover, disease-associated EVs serve as candidates for the targeted inhibition by pharmacological or genetic means. However, these attempts are often accompanied by major challenges, such as off-target effects, which may result in adverse phenotypes. This renders the clinical efficacy of EVs elusive, indicating that further understanding of the specific role of EVs in physiology may enhance their utility. This review highlights the essential role of EVs in maintaining cellular homeostasis under different physiological settings, and also discusses the various aspects that may potentially hinder the robust utility of EV-based therapeutics.

## 1. Introduction

Extracellular vesicles (EVs) are nanoparticles secreted by all cell types under various conditions. EVs are composed of a phospholipid bilayer, enclosing various proteins, lipids, nucleic acids and cellular metabolites, and, depending on the subtype, EVs may range from 30 to 10,000 nm in diameter [1]. Although most widely studied in mammalian cells, EV secretion occurs in species of all biological kingdoms, including cells harboring intact cell walls, such as yeast [2,3] and bacteria [4]. EVs facilitate paracrine and/or autocrine cell-to-cell communication [5,6] via the transfer of bioactive molecules. Currently, a limited number of studies emphasize the role of EVs in normal physiology [7,8]. This is due to the significance of EVs in various disease settings, which leads researchers to place a greater focus on the utility of EVs as potential therapeutics. Indeed, EVs obtained from various biological samples serve as non-invasive diagnostic biomarkers, since their cargo contents are highly reflective of the cells of origin [9,10,11,12]. Moreover, EVs are readily distributed throughout the body and, together with organotropism, EVs may be engineered for the targeted delivery of drugs and/or functional biomolecules to recipient cells with relatively high specificity [13,14,15,16,17].

Several studies indicate that the inhibition of EV secretion may also assist in preventing disease progression [16,18,19,20]. However, as EVs partake in numerous physiological processes, it is imperative to consider the potential risks when targeting EVs as a therapeutic strategy [21,22,23,24]. Indeed, it has been previously suggested that perturbations in the processes of EV biogenesis and/or secretion may hinder the maintenance of cellular homeostasis and ultimately disrupt the integrity of cells [25]. This further raises concerns regarding the benefits of employing EVs as therapeutics and the yet unknown implications of potential side effects. In this review, we provide a comprehensive overview of EV-mediated processes under normal physiological conditions. We also briefly narrate the implications of EVs in pathophysiological settings and potential complications that may be associated with EV-based therapeutics. Lastly, we summarize the outcomes for in vitro and in vivo models following the inhibition of genes implicated in EV biogenesis and secretion.

## 2. Background on EVs

### 2.1. Subtypes of EVs

**Figure 1:**
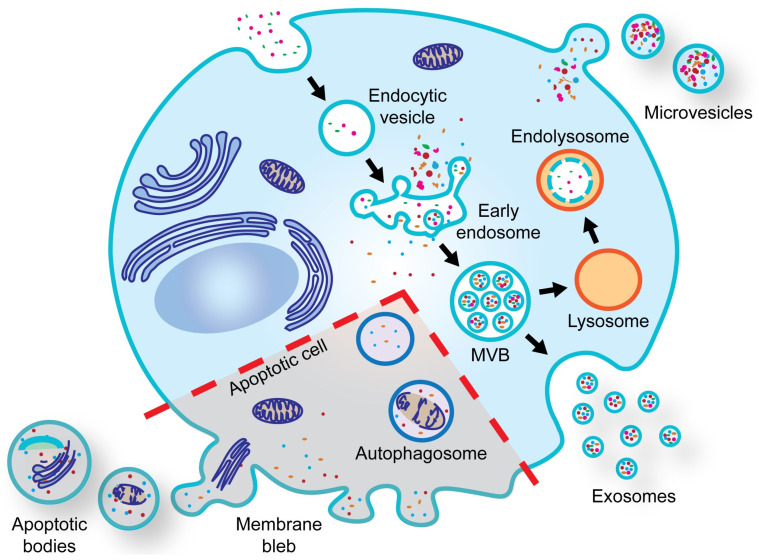
Biogenesis of extracellular vesicles. Evs are released by all cell types. The biogenesis of three major subtypes of Evs is shown. Exosomes are secreted via the endosomal pathway. In contrast, microvesicles originate directly from the plasma membrane, while apoptotic bodies are released from membrane blebs of cells undergoing apoptosis.

EVs are broadly segregated into small (<200 nm) and large (>200 nm) based on their size. However, due to the inherent overlap in sizes upon secretion, EVs can be further categorized based on the cellular pathways involved in their biogenesis [26]. Hence, a precise characterization defining the organellar origin of EV subtypes is recommended, as per the minimal information for studies of extracellular vesicles 2023 (MISEV 2023) guidelines [27]. Currently, the most widely studied subtypes of EVs consist of exosomes (small EVs) ranging from 30 to 150 nm, microvesicles (small/large EVs) ranging 100–1000 nm and apoptotic bodies (large EVs) ranging 1000–5000 nm in diameter [28]. In eukaryotes, such as mammalian cells, exosomes are synthesized via the endosomal pathway in an ESCRT-dependent or -independent manner (Figure 1). In brief, the invagination of early endosomal membrane allows for the sorting of biomolecules into intraluminal vesicles (ILVs). Subsequently, mature multivesicular bodies (MVBs) harboring ILVs fuse with either the plasma membrane (PM) to secrete ILVs (referred to as exosomes) or fuse with lysosomes for degradation [28]. Microvesicles are formed via the direct outward budding of the PM, whereas apoptotic bodies are formed during apoptotic cell disassembly, which triggers membrane blebbing in cells undergoing apoptosis [29,30].

**Figure 2:**
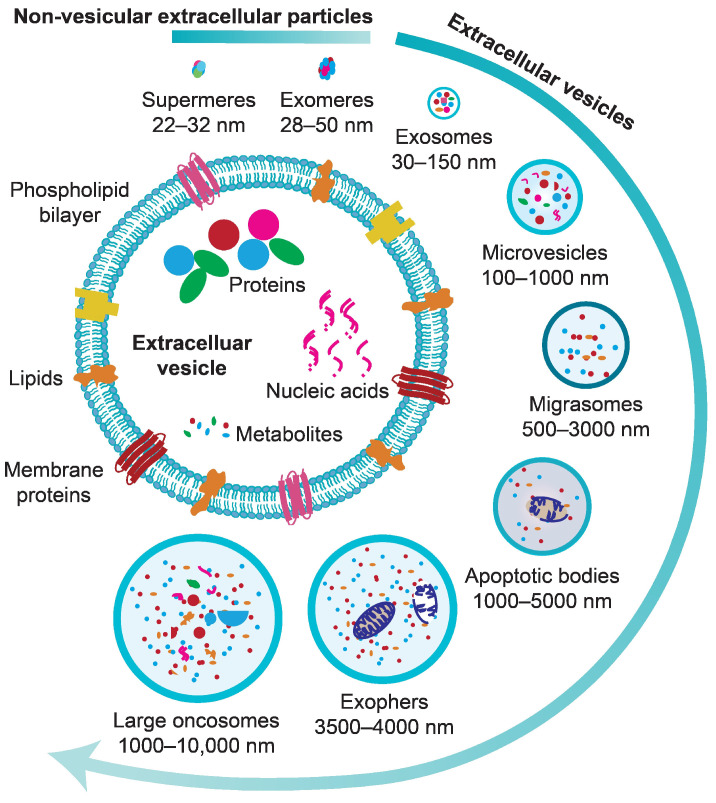
Diversity of extracellular vesicles and particles. EVs comprise a phospholipid bilayer enclosing various biomolecules as well as organelles, such as mitochondria, in larger EVs. Subtypes of EVs ranging from 30 to 10,000 nm in diameter are depicted. NVEPs, which are less than 50 nm in size, are also secreted by cells into the extracellular environment.

Recently, several other subtypes of EVs have been introduced (Figure 2), which include migrasomes, exophers and large oncosomes [31,32,33,34]. Further to this, several studies have identified the secretion of small, membrane-less, non-vesicular extracellular particles (NVEPs), such as exomeres and supermeres [35,36,37]. However, the exact mechanism of biogenesis, as well as the specific roles that the new subtypes of EVs/NVEPs play in physiological settings, remain to be further illustrated.

### 2.2. Characterization of EVs

The subtypes of EVs can be differentiated according to their unique characteristics. For example, EVs exhibit a distinct enrichment of proteins such as CD9, CD63, CD81, PDCD6IP (Alix), TSG101 and Flotillin [1,38]. The detailed guidelines for EV studies involving nomenclature, isolation, purity, functional characterization and data interpretation can be found in MISEV 2023 [27]. For example, EVs are routinely characterized by nanoparticle tracking analysis for size detection, EV-enriched proteins are examined via Western blotting and the morphology is visualized via microscopy techniques such as transmission electron microscopy. As mentioned previously, the secretion of EVs occurs in all known cell types within various tissues. Accordingly, the organ-specific secretion of EVs has been observed from the brain [39,40], liver [41], lungs [42], tumors [43], lymphoid tissues (spleen and lymph nodes) [44] and colon, among others [43,45]. While the general characteristics of these EVs remain consistent with one another, their functional characteristics may vary depending on the composition of bioactive molecules that closely resemble the tissues of origin [46].

EVs secreted by the aforementioned organs are abundantly present in bodily fluids (i.e., extracellular fluids), including blood [47], urine [48], cerebrospinal fluid (CSF) [49] and saliva [50], among others. However, the heterogeneity of EVs often creates difficulties in discerning the exact source and/or function of EVs that are present in complex biological fluids. Although various methods, namely differential-ultracentrifugation, size exclusion chromatography and flow cytometry, provide effective means of isolating EVs, obtaining a pure population of a single EV subtype or EVs derived from one particular tissue/organ is not yet achievable [51,52,53]. In spite of this notion, recent attempts have highlighted that the careful inspection of cell/tissue-specific markers that are enriched in EVs may assist in identifying their sources [54]. For example, renal-tubule-derived EVs, which are abundantly present in urine, are enriched in CD24 [55]. Similarly, EVs isolated from muscle cells display an abundance of proteins such as ATP2A1, β-enolase and desmin, which may serve as protein markers of skeletal muscle-cell-derived EVs [56,57]. Nevertheless, a more in-depth understanding of the biomolecular cargo within EVs, in addition to their size and morphology, may ultimately assist in uncovering the functional role of EVs present in complex biological fluids [58].

### 2.3. Cargo of EVs

As mentioned earlier, the biomolecular cargo of EVs is largely determined by the cells of origin [59,60,61,62]. Detailed information regarding the identified biomolecular cargo of EVs is currently available in publicly accessible databases such as Vesiclepedia (Table 1) [1]. In the recent years, the use of in vitro and in vivo assessments, including patient samples, has allowed for the expansion of knowledge concerning the various biomolecular profiles within EVs [63,64,65]. Studies further indicate the dynamic alterations in the functional cargo of EVs, which are often influenced by intrinsic and/or extrinsic factors such as temperature, hypoxia, exercise and diseases [57,66,67,68]. For instance, a study by Rayamajhi et al. highlighted that an increase in the incubation temperature of cells not only augments EV secretion but also results in the significant enrichment of Serpinb1, at least in EVs secreted by K7M2 mouse osteosarcoma cells. Rayamajhi et al. further depicted that cellular stresses induced by heat and nutrient deprivation trigger the increased detection of anti-proliferative and pro-proliferative proteins in EVs, respectively [68]. Similarly, exercise-induced EVs are enriched in various myokines as wells as proteins, such as RAB1A, ANXA2, ITGB1, ITGB2, ITGB5, Alix and FLOT1 [57]. The packaged cargo of EVs released by cancer cells have also been illustrated to change upon the administration of anti-cancer therapy, highlighting the utility of EVs as an indicator of prognosis for improved treatment efficacy [69]. In line with this, a recent study demonstrated that EVs isolated from lymphatic drainage exudate from breast cancer patients are enriched in CD24, CD29, CD44 and CD146, which are often considered potent cancer biomarkers [12]. These notions further emphasize that a clearer understanding of the precise EV contents is needed for their robust application as diagnostic biomarkers [12,70].

## 3. Role of EVs in Physiology

**Figure 3:**
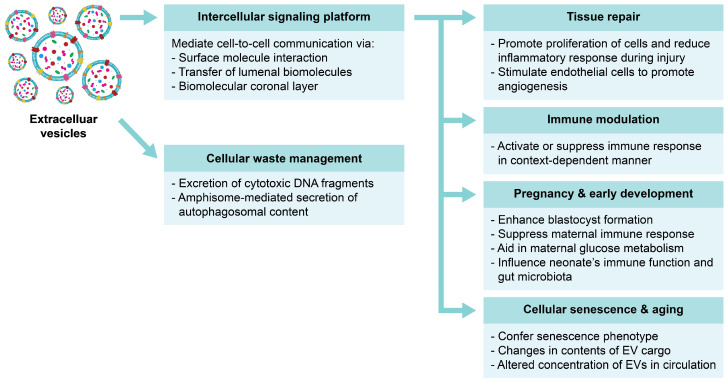
Various roles of EVs in normal physiology. EVs contribute to various aspects of normal physiological processes. The secretion of EVs by cells aids in the removal of cellular waste. Evidently, EVs also play a crucial role as mediators of intercellular communication to conduct biological processes such as tissue repair, the modulation of immune activity and aging, as well as promote healthy pregnancy and early development.

Although EVs are frequently highlighted as the key players orchestrating the processes involved in pathophysiology, emerging evidence continues to emphasize the significance of EVs in conducting normal physiological processes [71,72]. Examples of the implicated roles of EVs in normal physiology are depicted in Figure 3.

### 3.1. Cellular Waste Management

EVs were initially thought to be released from cells as a means of discarding cellular waste into the extracellular environment [73]. It is well established that intricate biological processes exist to regulate cytotoxicity within cells [74]. For example, the excessive build-up of cytosolic DNA fragments is counteracted by DNase II activity [75]. While the presence of DNA within EVs has been a prolonged topic of debate, a study by Takahashi et al. provided evidence that, in cells undergoing DNA damage, potentially harmful cytosolic DNA fragments are excreted from cells via EVs to achieve cellular homeostasis. [76]. Moreover, the depletion of EV biogenesis and secretion regulators, Alix and RAB27A, respectively, induces the cytosolic accumulation of chromosomal DNA, thereby activating the cytosolic DNA sensing machinery. This, in turn, triggers reactive oxygen species (ROS)-dependent DNA damage response in cells, leading to the induction of cell-cycle arrest or apoptosis [76,77,78].

Autophagy is a phenomenon that ensures the maintenance of cellular integrity via the degradation and recycling of bioactive molecules, which promotes the extended longevity of an organism [79,80]. As key members of the endomembrane system, the processes of autophagy and EV biogenesis share a common lysosomal degradation pathway [81]. To complement this notion, Hessvik et al. demonstrated that the inhibition of lysosomal fusion, mediated by the knockdown of phosphoinositide kinase PIKfyve, promotes the fusion of autophagosome and MVB to give rise to a hybrid endosomal organelle called an amphisome. It was further depicted that amphisomes readily fuse with the PM, where CD63-positive ILVs and p62-positive structures present within MVBs and autophagosomes, respectively, are simultaneously detected in the extracellular environment [82]. These findings suggest that the secretion of EVs actively prevents the intracellular accumulation of potentially cytotoxic material. Despite these notions, currently, there is a lack of evidence to illustrate the exact fate of EVs harboring potentially unneeded biomolecules upon exiting cells. However, at least in the case of viral infections, the detection of viral DNA in the cytoplasm triggers the host cells to readily secrete EVs packaged with viral DNA [76]. Once secreted, these EVs can interact with immune cells to stimulate anti-viral response, ultimately preventing viral propagation [83,84].

### 3.2. Intercellular Communication and Cell Signaling

Intercellular communication is mediated by a variety of cellular processes. These include cell-to-cell contact at cellular junctions and the secretion of biomolecules, directly or within EVs, into the extracellular environment [5,85]. EVs potently transport biomolecules to nearby cells or even to target cells residing in difficult-to-reach organs, such as the brain, by crossing the blood–brain barrier [86,87]. Hence, perhaps the most renowned function of EVs is their role in mediating intercellular communication. Indeed, many roles of EVs discussed in the current review are carried out through EV-mediated cell signaling via cell-to-cell communication. The regulatory functions of EVs are exerted in the recipient cells by either receptor–ligand interaction or the transfer of biomolecules following uptake [88]. A recent study highlighted that EVs mediate crosstalk between exercised muscle cells and liver through the transfer of differentially regulated proteins (e.g., glycolytic enzymes) to regulate glycolysis under conditions of high energy demand [57].

Conventionally, the communication between neuronal cells was thought to occur via the action potential, where neurotransmitters are directly released into synapses to regulate the activity of postsynaptic neurons [89]. However, in the last decade, EVs arose as important drivers of inter-neuronal communication [90]. In demonstration of this, Lachenal et al. depicted that differentiated cortical neurons readily secrete EVs in calcium- and glutamate-dependent manners [91]. Furthermore, upon stimulation by glutamate, both glial and neuronal cells of the central nervous system (CNS) secrete EVs, which are taken up by other neuronal cells with high specificity [92]. These findings suggest that the secretion of EVs correlates with the enhanced regulation of neuronal activity, at least in glutamatergic neurons.

As mentioned above, EVs play a pivotal role in regulating signaling pathways in the recipient cells. One such example is the modulation of Wnt signaling activity, which is a major pathway that governs the growth and survival of cells through regulating the effector, β-catenin [93]. Kalra et al. depicted that EVs are utilized as effective transporters of mutant β-catenin. Furthermore, the EV-mediated transfer of mutant β-catenin readily induces Wnt signaling activity in the recipient cells [94]. Similarly, Chairoungdua et al. illustrated that endogenous wildtype β-catenin is packaged and sequestered in EVs, thereby antagonizing excessive Wnt signaling activity in human embryonic kidney cells (HEK293T) [95]. In addition, the EV-mediated transfer of secretory and transmembrane ligands, such as Wnt, Hedgehog and Notch, initiates signaling pathways that are crucial during early development [96].

Interestingly, Beit-Yannai et al. proposed a novel inhibitory role of EV-mediated intercellular communication. It was depicted that, prior to the uptake of EVs, the recipient cells may also secrete EVs that bind and dimerize with donor EVs. This, in turn, attenuates EV uptake by the recipient cells, thereby evading donor EV-mediated modulation of signaling pathways [97]. Although the exact mechanism remains elusive, such a phenomenon may be crucial in regulating the non-specific modulation of various intrinsic signaling pathways to maintain cellular homeostasis.

It is believed that the function of EVs depends on the particular tissue/organ of origin as well as their status (e.g., healthy or diseased). For instance, microglia-derived EVs exert different functional outcomes in the recipient cells depending on whether the donor microglia reside in the cerebral cortex or in the spinal cord [98]. Similarly, EVs released from different bone constituent cells vary among one another in terms of composition [99] and function. For example, osteoclast-derived EVs are enriched in miR-214, which inhibits the function of the osteoblasts [100]. In contrary, osteoblast-derived EVs are enriched in RANKL, which is thought to promote the survival and function of osteoclasts [101]. Furthermore, a recent study showed that the normal human skin fibroblasts communicate with human umbilical vein endothelial cells (HUVEC) to promote wound healing and angiogenesis via EVs, potentially through the GSK-3β/β-catenin signaling activity. However, in the presence of high glucose levels, EVs potentially downregulate the GSK-3β/β-catenin signaling pathway, thereby hindering wound healing and angiogenesis [102]. Collectively, this evidence highlights the crucial functions of EVs as mediators of intercellular communication in different physiological settings and that these functions may vary depending on cell types, as well as the conditions from which EVs originate.

### 3.3. Immune Modulation

In the context of immune response, EVs largely display dual functionality. For example, while EVs are alluded as key players in orchestrating the maturation of immune cells as well as the activation of immune response via direct or indirect antigen presentation, they are also depicted as mediators of immunosuppression [103,104]. B lymphocyte-derived EVs contain peptides of MHC Class II that stimulate CD4+ T cells, suggesting that these EVs may be responsible for the modulation of long-term T cell memory [23]. Furthermore, EVs that are released by dendritic cells also consist of MHC class I and II, as well as other T cell stimulatory factors [105]. This indicates that EVs assist in eliciting immune response upon the activation of dendritic cells via presenting specific antigens to T-cells. This, in turn, stimulates B cells and augments the secretion of EVs to further aid in immune response. Contrastingly, EVs can also be utilized as promoters of anti-inflammatory response. For example, the administration of EVs to mice treated with doxorubicin (chemotherapeutic agent known to induce off-target cardiotoxicity) downregulates inflammasome and pyroptosis markers, such as TLR4, NLRP3 and caspase-1, IL1-β and IL-18, respectively, which results in reduced inflammation-associated pyroptosis. These EVs also decrease the levels of TNF-α and pro-inflammatory M1 macrophages, while enhancing the anti-inflammatory M2 macrophages, all of which culminate in alleviated cardiotoxicity [106]. In support of this, Pacienza et al. further depicted that the treatment of EVs isolated from human bone-marrow-derived mesenchymal stem cells (MSCs) onto macrophages from mice effectively prevents the lipopolysaccharide (LPS)-induced polarization of M0 into M1 macrophages to further enhance anti-inflammatory immune response [107].

Furthermore, EVs play a critical role in the prevention or control of infections. A recent study by Rausch et al. demonstrated the role of EVs in enhancing antiviral response of effector T cells against lymphocytic choriomeningitis virus in mice. It was shown that antigen-presenting cell-derived EVs interact with CD8+ T cells to activate T cell receptor signaling, which culminates in the enhanced proliferation of effector T cells [108]. Similarly, Emerson et al. suggested that EVs isolated from *Salmonella*-infected macrophages offer protection in mice subsequently infected with a lethal dose of the bacteria. The administration of these EVs effectively elicits anti-bacterial immune response by promoting the production of anti-OmpD IgA in mice [109]. Additionally, EVs released by *Mycobacterium tuberculosis* (*M. tb*)-infected neutrophils are potent activators of macrophages, promote the clearance of infection via enhanced superoxide anion production, and further induce autophagy in infected macrophages [110]. This evidence collectively illustrates the crucial immunomodulatory role of EVs, and that the lack of EV secretion by various cell types may adversely alter the immune system function in a context-dependent manner.

### 3.4. Tissue Repair and Angiogenesis

Evidently, MSCs have been implicated in cell/tissue differentiation during early development, and in tissue repair in later stages [111]. In particular, bone marrow-derived MSCs (BMSCs) aid in tissue repair via differentiating into organ-specific cell types, which, in turn, serve as substitutes for the damaged cells. BMSCs also secrete growth factors, prostaglandins as well as cytokines, and the administration of MSC-derived conditioned medium has been shown to provide protection in mice undergoing cisplatin-induced renal tubular damage [112]. In line with this notion, other studies further suggest that MSC-derived EVs are enriched in functional mRNAs that code for the regulators of cell cycle and proliferation, most notably IGF-1R. Moreover, the EV-mediated transfer of such mRNAs promotes the expression of cell cycle regulators, thereby conferring enhanced proliferation and resistance to apoptosis in mice undergoing glycerol/cisplatin-induced renal tubular injury [113,114,115]. Indeed, Kang et al. also demonstrated that human amniotic epithelial cell-derived EVs carry an mRNA signature that inhibits TNF-α/MAPK signaling-mediated apoptosis and inflammatory response, thereby alleviating symptoms of acute kidney injury [116]. Additionally, MSC-derived EVs promote cardiac repair in myocardial infarction rat models. Specifically, MSC-derived EVs are enriched in miR-29 and -24, which have previously been reported to suppress renal fibrosis and aortic vascular inflammation, respectively [117,118,119]. Similarly, a study by Nojima et al. showed that hepatocyte-derived EVs enhance the proliferative capacities of the recipient hepatocytes, and consequently promote liver regeneration in mice undergoing hepatic ischemia/reperfusion injury or partial hepatectomy. The observed effect is achieved via the EV-mediated transfer of neutral ceramidase, as well as sphingosine kinase 2, which was previously implicated in driving cell proliferation via the upregulation of MYC proteins [120,121].

Angiogenesis is considered a critical phenomenon in promoting tissue repair following injury [122,123,124], where EVs are often described as key mediators in orchestrating angiogenesis [125,126,127]. Kim et al. previously suggested that platelet-derived EVs harbor pro-angiogenic properties, which promote a dose-dependent angiogenic phenotype of HUVEC. Interestingly, the pro-angiogenic capacity of platelet-derived EVs diminishes upon the activated-charcoal-induced removal of lipid growth factors. This suggests that both protein and lipid growth factors, such as VEGF, FGF-2 and sphingosine 1-phosphate, respectively, are required for the acquisition of pro-angiogenic phenotype [128]. The pro-angiogenic properties of circulating EVs have also been linked to several tissue remodeling factors, such as matrix metalloproteinases (MMPs) [129]. Cavallari et al. previously demonstrated that endothelial cell-derived EVs isolated from human sera are enriched in MMPs (e.g., MMP-1 and -9), as well as other pro-angiogenic factors (e.g., TGF-β and angiogenin), and are capable of promoting vascular remodeling via the activation of MMPs and signaling pathways involving VEGF and TGF-β [130]. In support of this notion, another study further highlighted that the internalization of MSC-derived EVs that are enriched in VEGF and miR-210-3p enhances the proliferation, migration and formation of tube-like morphology in mouse endothelial cells, and further alleviates ischemic injury in mice [131]. The function of MSC-derived EVs in driving angiogenesis depends on oxygen availability to MSCs. For example, when comparing normoxic (18.4%) and physioxic (3%) conditions, MSC-derived EVs from physioxic conditions display an increased level of VEGF-A, hence eliciting a more noticeable pro-angiogenic effect via the augmented expression of FGF2, HIF1, VEGF and TGF-β in the recipient cerebral microvascular endothelial cells [132]. Additionally, cardiovascular progenitor cells derived from human pluripotent stem cells have also been shown to secrete EVs that improve the healing of cardiomyocytes upon myocardial infraction by promoting angiogenesis in mice [126]. Overall, these notions indicate that EVs serve as potent mediators of tissue remodeling and repair, and further hint at the potential utility of EV-based therapeutics for injury repair.

### 3.5. Pregnancy and Early Development

The development of embryos is dependent on the conditions of the maternal environment (e.g., nutrient availability). In order to achieve proper implantation and growth, a bidirectional maternal-to-embryo/fetal communication is crucial, where EVs have been implied to assist in the mentioned process [133]. Indeed, the EV-mediated transfer of RNA transcripts is believed to be critical in establishing the early stage of pregnancy by altering the endogenous RNA profile in the maternal endometrium [134]. For example, EVs derived from trophoblasts of viable embryos, but not degenerating embryos, carry RNA transcripts (e.g., *ZNF81* and *LTR7B*), and ultimately alter the expression of corresponding genes in the recipient endometrial cells [135]. In contrast, EVs secreted from the uterus of pregnant mice are enriched in miR-21 and are readily taken up by embryos upon implantation. The internalization of these EVs culminates in early embryonic stability as a result of enhanced blastocyst formation and the concomitant inhibition of apoptosis [136].

Throughout various stages of mammalian pregnancy, immunosuppression is critical in establishing a hospitable maternal environment [137]. In support of this, emerging evidence shows that human placenta explant-derived EVs harbor MIC and ULBPs. These EV-bound ligands subsequently attenuate immune cell-induced cytotoxicity, following the downregulation of NKG2D, which is a known receptor of natural killer (NK) and T cell activation [138]. Placenta-derived EVs are also enriched in placenta-specific phosphatases, known as PLAPs. The size of the PLAP-positive population of EVs detected within the total circulating EVs from maternal/fetal plasma is believed to be positively correlated with normal birth weight. This suggests that PALP-positive EVs may serve as an indicator of healthy pregnancy, and simultaneously as an early diagnosis marker of abnormal pregnancies (e.g., fetal growth restriction and small gestational age) [139].

Interestingly, Buca et al. highlighted that there are variations in the concentrations of EVs that originate from different cell types at different stages of pregnancies. For example, although the concentrations of EVs derived from either leukocytes or endothelial cells remain unchanged, higher concentrations of EVs derived from platelets and epithelial cells are observed at the first and third trimester, respectively [140]. This observation indicates that EVs secreted by various cell types may assist in ensuring the progression of healthy pregnancy. In support of this notion, existing evidence also illustrates the role of circulating EVs in regulating maternal glucose homeostasis. For example, human endometrial stem cell (hESC)-derived EVs actively transfer GLUT1 to the recipient ESCs, and promote decidualization via enhancing glucose uptake as well as the upregulation of IGFBP1, HAND2 and PRL [141]. Zierden et al. also indicated that factors such as glucose and insulin levels trigger an increase in the average concentrations of circulating EVs in pregnant mice. Moreover, the increased level of placental glucose-sensing enzyme, O-glycosyl transferase, is positively correlated with circulating EV concentrations to aid in efficient glucose metabolism during pregnancy [142].

Upon birth, the early development of an infant is highly dependent on nutrients provided by the mother. Mammalian breast milk has been depicted as a rich source of EVs that are associated with positive outcomes, for example, promoting the growth and survival of intestinal epithelial cells to prevent necrotizing enterocolitis in neonates [143,144]. It is widely accepted that the ingestion of breast milk introduces immune-modulatory components to the offspring. This further allows for the healthy development of the intestinal immune system and establishment of gut microbiota [145]. Evidently, the proteomic profile of breast-milk-derived EVs shows the enrichment of proteins with an immune cell origin [146]. Furthermore, alterations in the type as well as the abundance of breast-milk-derived EV cargo, such as human milk oligosaccharides, have been illustrated to dynamically influence the immunity and gut microbiota of infants throughout different phases of lactation, as previously reviewed elsewhere [147]. Colostrum-derived EVs are also implicated in regulating the immune system and stimulating the growth of infants [148]. Conversely, when compared to that of non-diabetic mothers, EVs isolated from breast milk of type 1 diabetic mothers display differentially regulated miRNA profiles. For example, EVs present in the breast milk of type 1 diabetic mothers are enriched in miR-4497 and -3178, which elevate the production of pro-inflammatory cytokines, such as TNF-α, by macrophages upon LPS stimulation [149]. Interestingly, humans continue to consume milk from other species into adulthood. While this is a controversial topic of debate, the collective evidence suggests that EVs present in bovine milk are readily bioavailable to humans upon ingestion, and further ameliorate the growth of cells via the upregulation of miR-21-mediated mTORC-1 signaling [150,151]. Overall, these observations suggest that EVs play a pivotal role throughout mammalian pregnancies and early development, and also that exposure to EVs that are sourced from other organisms may be beneficial, although further investigation is required to support this notion.

### 3.6. Cellular Senescence and Aging

The physiological processes involved in aging are largely attributed to cellular senescence, which is a phenomenon that is characterized by the induction of reversible or irreversible cell cycle arrest [152,153]. It is well-established that cells undergoing cellular senescence exhibit a concomitant increase in the secretion of EVs. Notably, normal human dermal fibroblasts undergoing senescence display a 15-fold increase in the amount of secreted EVs [154]. Existing evidence also suggests that, when compared to young donors (aged 29–36), plasma samples from elderly donors (aged 70–92) display increased abundances of EVs. Further to this, plasma-derived EVs from elderly donors confer a senescence-like phenotype to neighboring cells, which is believed to be partly mediated via the transfer of IFITM3 [155]. Contrastingly, a study by Eitan et al. demonstrated that there is a negative correlation between the amount of EVs in circulation and aging. In addition to the changes in the cargo composition of EVs, blood samples from elderly donors exhibit a reduction in the number of EVs when compared to those of young donors. Such a reduction in the number of circulating EVs is attributed to the rapid internalization of EVs by B cells as well as monocytes, but not by T-cells [156]. In line with this notion, another study demonstrated that, when compared to the young ones (aged 3-months), older Wistar rats (aged 21 or 26 months) display a notable decrease in the amount of circulating EVs, as evidenced by the reduced CD63 abundance. Moreover, it was shown that circulating EVs isolated from older rats contain an increased level of ROS. In light of this, the authors further explained that aerobic exercise, although temporarily, reverts the observed phenotypes [157], to highlight the involvement of EVs in healthy aging process.

Existing evidence also suggests the occurrence of significant alterations in the cargo contents within EVs that are secreted by aged cells. One such example is the changes in the detected abundance of the brain-derived neurotrophic factor (BDNF) and proBDNF (BDNF precursor). It is widely recognized that BDNF is involved in the development and protection of the nervous system, while proBDNF is often involved in the induction of neuronal apoptosis [158,159]. Indeed, proBDNF, which has previously been associated with memory loss in older mice, has also been shown to be highly enriched in human-plasma-derived EVs from older individuals [158,160]. Suire et al. demonstrated that, in L1CAM (neuronal marker)-positive EVs from plasma samples of older individuals, significantly increased levels of proBDNF are detected, which is believed to be correlated with older individuals’ decline in walking speed [160]. Similarly, elevated miR-185-5p level has also been reported in BMSC-derived EVs present in the interstitial fluid of aged mice. The subsequent uptake of miR-185-5p-enriched EVs leads to senescence-like features in BMSCs, and further downregulates osteogenic properties [161]. Contrastingly, when compared to that of older rats, EVs isolated from sera of young Wistar rats display an enrichment of miR-219, which is a pro-myelinogenic factor involved in promoting healthy functions of the CNS. Interestingly, upon environmental enrichment, aged rats also exhibit increased levels of miR-219 within the secreted EVs [162]. These notions collectively suggest that alterations in EV cargo and the amount of circulating EVs are implicated in age-related degeneration, and also serve as indicators of healthy aging.

## 4. Role of EVs in Pathophysiology

EVs are highly implicated in pathological conditions such as cancer [163,164,165]. For example, various cancers utilize EVs as a means to communicate with distant skeletal muscles and adipose tissues to induce muscle wasting and lipolysis, respectively, and triggers the onset of cancer-associated cachexia [166]. Furthermore, the internalization of EVs that are derived from chemoresistant cancer cells confer chemoresistance to neighboring cancer cells, at least in neuroblastoma and pancreatic cancer [167,168]. EVs are also highlighted as important mediators of cancer metastasis by augmenting the establishment of pre-metastatic niche prior to the invasion of tumor cells [169]. Indeed, Peinado et al. showed that EVs secreted by melanoma cells assist the conversion of bone marrow progenitor cells into cancer-specific stromal cells [19]. The organotropism of primary tumor-derived EVs in various cancers often involves the recognition of surface markers present on EVs by the recipient cells. For example, EVs released from metastatic breast cancer cells are enriched in various integrins, which assist in the adhesion of EVs to the recipient cells [170]. Moreover, this receptor–ligand interaction depends on the types of integrins present on the surface of EVs to allow for selective uptake to occur, and ultimately enhances the organotropic properties of EVs [16].

Contrastingly, existing evidence suggests that soluble factors that are present within biological fluids, such as blood and interstitial fluids, may facilitate surface interaction with EVs to give rise to the protein corona (PC) surrounding EVs [171]. The EV-bound PC also exhibits dynamic alterations in its biomolecular composition depending on the physiological conditions. For example, when compared to that of healthy subjects, breast cancer patient-derived EVs are highly associated with cytokines, such as CCL2, which promote the establishment of a metastatic niche [172]. Despite this notion, the exact implications concerning the presence of PC interfering with the biodistribution, as well as the therapeutic efficacy of EVs, require further investigation [173].

Tumor-derived EVs drive cancer progression by promoting the invasive capacities of cancer cells [174]. Indeed, the uptake of CEMIP-enriched EVs secreted by brain-tropic breast cancer cells subsequently triggers brain endothelial cells to exhibit a tube-like morphology, and accelerates the formation of pre-metastatic niche [175]. A similar effect of EVs has been observed in various cancers, such as pancreatic, ovarian, colorectal, renal, gastric and hepatocellular, as well as head and neck squamous carcinoma [176,177,178,179,180], suggesting the enhanced angiogenic properties of EVs in driving tumorigenesis. This phenomenon can be attributed to the activation of Egr-1 via ERK1/2 and JNK signaling, at least in colorectal adenocarcinoma [181]. In contrast, the internalization of EVs secreted by MSCs derived from menstrual blood exhibit diminished angiogenic and proliferative capacities in oral squamous cell carcinoma [182]. Similarly, NK cell-derived EVs are enriched in cytokines and chemokines, and consequently exhibit cytotoxicity in melanoma and neuroblastoma cells [183,184]. A study by Choo et al. highlighted that M1 macrophage-derived EVs contain pro-inflammatory cytokines, and readily repolarizes M2 tumor-associated macrophages into M1 macrophages. In addition, M1 macrophage-derived EVs act synergistically with anti-PD-L1 to enhance the efficacy of immune checkpoint inhibitor therapy [185]. Milk-derived EVs have also been shown to possess anti-proliferative properties in neuroblastoma, as well as anti-metastatic effects in breast cancer, although in a context-dependent manner [15,186]. This evidence collectively highlights that EVs play anti- or pro-tumorigenic roles depending on the context.

In addition to cancer, previous findings also suggests that EVs are highly implicated in diseases such as neurodegenerative diseases as well as various infectious diseases [187,188,189]. In Alzheimer’s disease, it has been shown that microglial cells readily secrete EVs that trigger the perturbed morphology of neuronal dendrites, which then promote synaptic dysfunction via the transfer of amyloid-β [190]. Moreover, D’Acunzo et al. also described that mitovesicles (double-membraned electron-dense EVs of mitochondrial origin [191]) derived from the brains of a Down syndrome mouse model hinder the synaptic activity (i.e., long-term potentiation) of hippocampal neurons via the transfer of monoamine oxidase B [192]. As mentioned previously, autophagy plays a crucial role in extending the longevity of cells, particularly in neuronal cells [80,193]. Indeed, defective autophagy has been implicated in Huntington’s disease, where neuronal cells lack the ability to degrade aggregates of HTT proteins that are composed of polyglutamine (polyQ) residues [194]. Interestingly, a recent study introduced a potential neuroprotective role of EVs in assisting in the clearance of polyQ aggregates. Yang et al. demonstrated that the inhibition of early-stage autophagy genes, excluding *atg-16.2*, triggers the secretion of exophers from neuronal cells of *Caenorhabditis elegans* to promote the excretion of polyQ aggregates, which consequently decreases the intracellular accumulation of polyQ aggregates [195].

It is currently understood that viruses often hijack the pathways involved in EV biogenesis and secretion for viral propagation. The enclosure of mature viruses in EVs allows for the evasion of host immune system and exacerbates viral infections [196,197,198]. A recent study by Kumari et al. highlighted the role of EVs in mediating viral infection, particularly in dengue. It was shown that plasma EVs derived from patients suffering severe dengue display significantly elevated levels of pro- and anti-inflammatory cytokines, such as IFNγ, TNF-α and IL-13, respectively. These EVs also trigger a reduction in recipient T cell proliferation as well as apoptosis, and ultimately suppress CD4+ T-cell activation via the increased expression of PD-1 on the surface of T cells [199]. Similarly, EVs released from poly(I:C)-treated respiratory epithelial cells (i.e., induced antiviral response) contain an abundance of immunomodulatory enzyme, PKM2, which impairs the recipient macrophage function, and fail to provide protection against secondary infection with *Staphylococcus aureus* [200].

It is also well documented that a host of virulence factors are secreted within EVs to aid in the propagation of bacterial infections [201,202]. For example, Prados-Rosales et al. previously described that *M. tb* as well as *M. bovis* readily secrete membrane vesicles (MVs) to mediate host–pathogen interactions. MVs secreted by *M. tb* and *M. bovis* contain an abundance of lipoproteins that serve as TLR2 signaling agonists. Moreover, the administration of *M. bovis*-derived MVs to mouse bone marrow-derived macrophages potently initiates the production of pro-inflammatory cytokines, including various interleukins (e.g., IL-1β, -6, -10 and -12), as well as CXCL1 and CCL3, in a TLR2 signaling-dependent manner [203]. In line with this notion, Schirmer et al. further illustrated that *M. tb*-derived MVs present particular immunogenic antigens, which may serve as potential diagnostic markers of tuberculosis [204].

## 5. Potential Complications in Establishing EV-Based Therapeutics

EVs are relatively stable under harsh conditions, and the presence of a lipid bilayer consequently provides protection to the biomolecular cargo within [205,206]. This encourages EVs to be investigated as a potentially ideal mode of delivering therapeutic agents to target cells [207]. Upon administration, EVs are readily distributed throughout various organs. For instance, the administration of milk-derived EVs via oral gavage successfully delivers proteins of bovine origin to the livers of mice [15,208]. Furthermore, the literature suggests that packaging of drugs within EVs minimizes drug-mediated cytotoxicity during therapy [13]. A recent study also demonstrated that platelet-derived EVs, generated via extrusion, sonication or freeze/thaw, can be used as carriers of doxorubicin. Moreover, the administration of EVs packaged with doxorubicin potently induces anti-cancer effects in MDA-MB-231 breast cancer cells, and to a lesser extent in another breast cancer cell line, MCF7 [209].

Despite these notions, the question of the target cell specificity of EVs in a complex biological system still remains. Although illustrated in the context of cancer metastasis, Hoshino et al. provided the insight that EVs derived from various cancer cells with metastatic potential display varying abundances of integrins, which allow for preferential interaction with specific target cells [16]. This observation suggests that the modifications of surface markers present on EVs that are engineered for therapeutic purposes may be utilized to target specific organs to avoid potential off-target effects. Additionally, it can also be deduced that a more in-depth understanding of EV surface markers and the effective inhibition of cancer-associated EV-to-cell interaction may potentially alleviate the disease burden, at least in metastatic cancers [18]. Indeed, it has been suggested that the aberrant regulation of genes implicated in EV biogenesis and secretion displays positive correlations with diseases such as degenerative polyarthritis and squamous cell carcinoma, among others [210]. Nevertheless, the potential use of EVs in therapeutic settings, either as a vehicle of drug delivery or via hindering the secretion of disease-associated EVs, requires further assessment [13,211,212], and some of the concerning factors that may accompany EV-based therapeutics are discussed below.

### 5.1. Coagulation and Immunogenicity

Currently, various pre-clinical studies involving EVs isolated from diverse sources are actively being conducted by independent groups. Despite this collective effort, the appropriate concentrations of EVs (measured by the number of particles, amount of protein or amount of compound packaged in EVs per kg of body weight) prepared for administration via various routes of entry, as well as their precise pharmacodynamics and pharmacokinetics, are unclear, as previously discussed by Gupta et al. [213].

Intravascular injection is one of the common administration routes to achieve the efficient delivery of drugs [214]. Similarly, numerous studies involving the administration of EVs routinely introduce EVs via intravascular injection in an attempt to uncover the relevant physiological roles of EVs. Although a large body of evidence currently suggests successful observational outcomes concerning the phenotypes in question, findings in other studies imply potential risks, such as coagulation and thrombosis, that may be associated with the intravascular administration of EVs [215,216,217,218]. Berckmans et al. described that EVs secreted by various cells, likely granulocytes and epithelial cells, accumulate in saliva in the body. These EVs are enriched in intravascular tissue factors (TFs) that accelerate coagulation upon contact with blood via enhanced factor VII-mediated clotting [216]. Similarly, independent studies also reported the TF- and thrombin-dependent pro-coagulant activity of EVs in augmenting platelet aggregation and potentially promoting thrombogensis [215,217]. Further to this, another study by Silachev et al. simulated that EVs isolated from human umbilical cord MSCs also harbor pro-coagulant properties that are imposed by TF and phosphatidylserine present on the surface of EVs. Indeed, the addition of MSC-derived EVs to whole blood and platelet-depleted plasma from healthy individuals elicits 9- and 4-fold increases in coagulation, respectively [219].

Similarly, while many pre-clinical studies indicate that the administration of EVs is likely to exhibit low immunogenicity, others imply that more comprehensive assessments that narrate the varieties in the cell-type-specific cargo of EVs are required [220,221]. EVs carry a host of immunoregulatory molecules on their surface, and therefore serve as potent modulators of immune activity [222]. It is also recognized that various cell types, including immune cells themselves, secrete EVs that regulate immunogenic response [104]. For example, T regulatory cells, cultured ex vivo, secrete EVs that effectively suppress pro-inflammatory response in mice stimulated with LPS [223]. Moreover, EVs isolated from MSCs of the fetal liver present TGF-β on their surface, which, in turn, impairs the function of NK cells via the activation of Smad pathway [224]. On the other hand, circulating EVs isolated from mice undergoing sepsis contain various miRNAs (e.g., miR-122 and -146a), which aid in eliciting TLR7- and MyD88-mediated pro-inflammatory response in mice [225].

Currently, HEK293T cells are among the commonly employed cell lines as a rich source of potential therapeutic EVs [226]. Indeed, Zhu et al. previously indicated that a treatment involving a low dosage (8.5 μg of protein) for a short duration (three times per week for three weeks) of HEK293T-derived EVs does not induce a visible immune reaction in mice. However, the authors simultaneously noted that the potential implications of treatments involving a higher dosage for a longer duration in eliciting an immunogenic response remain unknown [227]. In line with this notion, a comprehensive characterization of biomolecular cargo conducted by Li et al. identified the presence of oncogenic as well as immunogenic molecules (e.g., epidermal growth factor receptor, Src and Raf kinases) in HEK293T-derived EVs [228]. Further to this, Fitzgerald et al. demonstrated that EVs isolated from various sources, including biological fluids, cultured tissue explants and immune cells, encapsulate soluble cytokines that remain undetected using conventional cytokine assays [229]. Overall, the information currently described in the literature suggests that a clearer understanding of EV-enriched cargo derived from various sources is required to eliminate the potential side effects of EV-based therapeutics.

### 5.2. Pharmacological Inhibition of EV Biogenesis and Secretion

An alternate approach to the use of EVs in a therapeutic setting is to modulate the biogenesis and secretion of disease-associated EVs [230,231]. In attempts to achieve this, several drugs have been identified that effectively inhibit the biogenesis and secretion of EVs [230]. GW4869 is a potent inhibitor of neutral sphingomyelinase (nSMase) and is widely used to study the functional roles of EVs by suppressing biogenesis [232,233]. However, a study by Tabatadze et al. demonstrated that the inhibition of nSMase-2 by GW4869 negatively affects spatial memory formation via the perturbation of synaptic activity in mice [234]. Moreover, nSMase-2 has been implicated in the activation of p38 MAPK via the upregulation of ceramides in the Golgi. This inhibits mTOR signaling, and induces autophagy to counteract against starvation and nutrient depletion, which further highlights the significance of nSMase-2 in a biological system [235]. Considering these notions, targeting nSMase activity may prove detrimental to cells under normal conditions or during a nutrient-deficient state. On another note, the antibody-directed inhibition of tetraspanins, CD9 and CD63, has been shown to result in a decline in breast cancer-derived EVs in circulation, ultimately alleviating cancer progression in mice [236]. In line with this, McNamee et al. described several other drugs (e.g., calpeptin, Y27632 and manumycin A) that effectively hinder the secretion of EVs and further attenuate the migration of triple-negative breast cancer cells [18,237]. The advancements in high-throughput screening allowed for the further identification of drugs such as tipifarnib and ketoconazole as potential inhibitors of EV biogenesis [238]. Similarly, drugs such as Nexinhib20 and Y27632, have also been illustrated as inhibitors of RAB27A- and ROCK-mediated EV secretion, respectively [239,240]. However, despite the identification of various drugs, the complete pharmacological inhibition of EVs has not been achieved, and the implications of the complete inhibition of EV secretion in a biological system remain elusive [237,241].

### 5.3. Genetic Modification of Genes Implicated in EV Biogenesis and Secretion

Perhaps a more widely utilized method of modulating the biogenesis and secretion of EVs is through the depletion of genes that are involved (Table 2). TSG101 is one of the most commonly employed protein markers for the characterization of EVs, as it serves as a key mediator of EV biogenesis [242]. TSG101 is also a tumor-suppressor that is involved in regulating cell growth [243]. Given that TSG101 partakes in various cellular phenomena, targeting TSG101 in an attempt to inhibit the biogenesis of EVs may be detrimental to cellular homeostasis [244]. An in vitro study previously highlighted that the depletion of TSG101 in cells is implicated in endoplasmic reticulum (ER) remodeling and the lysosomal degradation pathway, thereby resulting in ER stress [245]. Further evidence denotes that the conditional WAP-Cre-based knockout of TSG101, specifically targeted to differentiate mammary epithelial cells in mice, triggers early embryonic lethality [246]. Moreover, the loss of TSG101 induces MDM2/p53-mediated G_1_ cell cycle arrest and increases cell death in mouse mammary epithelial cells, which consequently imposes the loss of the ability to lactate through the abolished development of mammary glands [246,247]. Similarly, another key regulator of EV biogenesis, Alix, also governs apical–basal polarity in epithelial cells via the maintenance of the actomyosin–tight junction relationship. As such, the depletion of Alix in mice has been implicated in severe epithelial structural defects, particularly in the choroid plexus and ependyma, giving rise to hydrocephalus [248]. Understandably, Alix is highly implicated in normal development and brain growth in mice [249], where endocytosis at brain synapses is mediated by Alix for correct synaptic physiology [250].

nSMase2 is also a well described target when combatting several diseases in which EVs play a role [251]. A recent study highlighted that as nSMase2 activity is crucial for HIV replication and propagation, targeting the nSMase2-medicated biogenesis of EVs may be beneficial against HIV infection. Indeed, the knockdown of nSMase2 was further depicted to promote cell death in HIV-infected cells [252]. While the inhibition of nSMase2 has also been shown to effectively reduce EV secretion, studies also indicate the occurrence of context-dependent and contrasting phenotypes. For example, mice suffering from atherosclerosis benefit from mutation-induced deficiencies in nSMase2, most notably via alleviated plaque accumulation and vascular lesions [253]. On the other hand, the in vitro knockdown of nSMase2 in cardiosphere-derived cells downregulates features associated with EV-mediated cardiac repair [254]. Moreover, the loss of nSMase2 exhibits postnatal dwarfism caused by dysregulated ossification in mice [255].

Although the exact mechanism is not yet fully understood, tetraspanins are highly implicated in EV biogenesis [256]. For example, the knockout of CD63 in HEK293 cells in vitro triggers the reduced secretion of small EVs [257]. Furthermore, the existing evidence suggest that although no significant alteration in the endosomal compartments was observed in CD63-deficient embryonic fibroblasts derived from mice, these mice exhibit symptoms of diuresis, evident via an abnormal increase in water intake, which is accompanied by an increased urinary flow and reduced urine concentration [258]. Given the mildly adverse phenotype, CD63 may serve as a fruitful target for the inhibition of EVs as a potential therapeutic strategy. However, a more recent study by Tognoli et al. proposed that the siRNA-mediated knockdown of CD63 does not alter the release of EVs, at least in MDA-MB-231 cells [259]. Moreover, further evidence highlighted that the double knockout of CD63 along with CD81 accelerates ageing and reduces the lifespan in mice [260]. CD81 has also been identified as a key player in energy homeostasis. Accordingly, the complete removal of CD81 in mice has been implicated in obesity and adipose tissue inflammation, as well as insulin resistance [261].

Rab GTPases are often involved in the process of MVB-to-PM docking to mediate the secretion of EVs into the extracellular space [262]. Existing evidence illustrates that RAB27A is highly upregulated in differentiating osteoclasts. Indeed, the siRNA-mediated knockdown of RAB27A leads to the enlargement and multinucleation of osteoclast progenitor macrophages and further triggers defects in the localization of lysosomes [263]. In contrast, RAB27A knockdown in differentiated osteoclasts improves the pro-osteogenic function of osteoblasts upon ovariectomy in mice by suppressing the secretion of EVs harboring miR-214 [100]. *Ashen* mice are widely used to reflect on the function of RAB27A due to the lack of endogenous expression [264]. Deficiencies in functional RAB27A have been previously implicated in choroideremia, predominantly in males [265]. Moreover, various cell types derived from *Ashen* mice (e.g., acinar cells, melanocytes and T lymphocytes) display primary defects in the trafficking of endosomal vesicles and granular structures. Such defects culminate in the reduced pancreatic secretion of digestive enzymes, reduced density of platelet containing granules and increased cytotoxicity of T-cells [264,266,267]. Interestingly, previous findings suggest that the inhibition of various Rab GTPases may result in a cell-type-dependent response in altering the secretion of EVs. For example, while the knockdown of RAB11 in *Drosophila melanogaster* S2 cells reduces the secretion of EVs, no change is detected in HeLa cells, as reviewed elsewhere [268]. In a study by Ma et al., RAB27A was found to be an important regulator of EV secretion in the brain, which further provides protection against cerebral ischemia in an ischemic stroke mouse model. Brain-derived EVs obtained from RAB27A knockout mice also fail to attenuate ischemic injury in vivo, which implies that the RAB27A-dependent secretion of EVs may be critical to brain functioning [269]. Another study revealed that the siRNA-mediated knockdown of RAB27A attenuates hair growth in vitro [270]. Perhaps more importantly, Kren et al. demonstrated that the knockdown of RAB27A in pancreatic cancer cells effectively triggers epithelial-to-mesenchymal transition, and consequently promotes the invasive phenotype, at least in the early stage of metastasis [271].

**Table 2:** Summary of research findings involving modifications of genes implicated in EV biogenesis and secretion.

Gene	Modification	Model	Phenotype	Reference(s)
CD9	Whole-body knockout	C57BL/6J mice	Infertility	[272]
CD63	Whole-body knockout	C57BL/6J mice	Water diuresis	[258]
CD63	Knockdown	Zebrafish	Inability to hatch	[273]
CD81	Whole-body knockout	C57BL/6 mice	Reduced humoral immune response	[274]
CD81	Whole-body knockout	129/SvJ mice NIHS-BC/Tac mice	Brain enlargement	[275]
CD81	Whole-body knockout	FVB mice	Obesity Insulin resistance	[261]
PDCD6IP	Whole-body knockout	C57BL/6 mice	Hydrocephalus	[248]
TSG101	Whole-body knockout	C57BL/6C mice	Embryonic lethality	[246]
TSG101	Conditional knockout	C57BL/6C mice	Undeveloped mammary gland	[246]
TSG101	Knockdown	Zebrafish	Embryonic lethality	[276]
SMPD3	Whole-body knockout	C57BL/6 mice	Postnatal dwarfism	[255]
RAB27A/B	Whole-body knockout	C57BL/6 mice	Low-grade inflammation	[277]
RAB27A	Whole-body knockout	C3H/HeSn-*ash*/*ash* mice	Increased bleeding duration	[267]
RAB27A	Whole-body knockout	C3H/HeSn-*ash*/*ash* mice	Reduced T-lymphocyte cytotoxicity	[266]
CTTN	Whole-body knockout	C57BL/6 mice	Embryonic lethality	[278]

Another important aspect to consider while conducting a partial or complete depletion of genes is the presence of a compensatory mechanism. The occurrence of this phenomenon may potentially attenuate the desired phenotypic outcome in order to restore the balance within a biological system [279]. In the context of EV secretion, it has been previously shown that the knockdown of RAB27A has also proven inefficient in hindering the secretion of EVs due to the simultaneous overexpression of endogenous Cortactin [280]. This highly intelligent cellular mechanism further suggests that EV secretion is a complex and coordinated process involving various key players, including RAB27A, Coronin 1B and Cortactin, among others [281]. Therefore, a thorough assessment concerning the validity of the targeted inhibition of EV secretion must be considered to conclude its feasibility in a therapeutic setting.

## 6. Concluding Remarks

Although the functional outcomes imposed by the secreted EVs may vary depending on the context, EVs continue to play significant roles in both the normal and pathophysiological processes. To this day, there exists a comparatively smaller body of evidence elaborating the precise role of EVs in healthy conditions. Hence, the current review attempted to provide a broader insight into a number of physiological processes that EVs are currently implicated in. Notably, EVs ensure homeostatic balance by mediating intercellular communication/signaling in an effort to orchestrate biological processes such as tissue repair and immune modulation. This further implies that the loss of major players involved in the biogenesis and secretion of EVs may trigger potentially detrimental outcomes that hinder the normal functions of the body. Currently, major emphasis in the field of EV research is placed on unveiling the role of EVs and their therapeutic potentials in a variety of diseases. For example, EVs facilitate multiple aspects of cancer, such as the enhanced metastasis and conferral of chemoresistance, to ultimately aid in tumorigenesis and cancer progression [19,168]. It is evident that the pharmacological and/or genetic suppression of cellular processes involved in the biogenesis and secretion of disease-associated EVs (e.g., tumor-derived EVs) is likely to result in improved prognoses.

Additionally, existing pre-clinical studies also provide positive indications regarding the delivery of drugs and/or biomolecules via EVs. However, despite the current depth of knowledge, the robust utilization of EVs as therapeutic targets of inhibition or drug delivery has not yet come to fruition. This is largely due to the inherent accompaniment of still undisclosed complications involving the disruption of normal cell/tissue homeostasis, as well as the incomplete understanding of the precise properties of EV cargo arising from various sources. Furthermore, to fully harness the therapeutic potentials of EVs, an establishment of guidelines that specify various aspects, such as the purity and storage conditions, of the isolated EVs, as well as the potential side effects, appropriate dosages and treatment duration, and the precise pharmacokinetics and pharmacodynamics, are imminently required. Overall, a clearer picture of the specific functions of EVs and their cargo in both the normal and pathophysiological functions will surely open new avenues to fully utilize the potentials of EVs in various clinical settings.

## Figures and Tables

**Table 1:** List of the top 15 most identified proteins in EVs recorded in Vesiclepedia. Protein names and their respective molecular weights are as denoted in the UniProt database.

Rank	Gene Symbol	Protein Name	Predicted MW (kDa)	Number of Entries in Vesiclepedia
1	CD63	CD63 antigen	25.637	1119
2	CD9	CD9 antigen	25.416	972
3	PDCD6IP	Programmed cell death 6-interacting protein	96.023	884
4	TSG101	Tumor susceptibility gene 101 protein	43.944	847
5	CD81	CD81 antigen	25.809	704
6	GAPDH	Glyceraldehyde-3-phosphate dehydrogenase	36.053	593
7	FLOT1	Flotillin-1	47.355	549
8	ACTB	Actin, cytoplasmic 1	41.737	538
9	ANXA2	Annexin A2	38.604	514
10	SDCBP	Syntenin-1	32.444	481
11	HSP90AA1	Heat shock protein HSP 90-alpha	84.660	457
12	HSPA8	Heat shock cognate 71 kDa protein	70.898	453
13	ANXA5	Annexin A5	35.937	447
14	ENO1	Alpha-enolase	47.169	441
15	PKM	Pyruvate kinase PKM	57.937	436

## Abbreviations

BDNF	brain-derived neurotrophic factor
BMSC	Bone marrow-derived mesenchymal stem cell
CNS	Central nervous system
ER	Endoplasmic reticulum
ESC	Endometrial stem cells
ESCRT	Endosomal sorting complex required for transport
EV	Extracellular vesicle
HEK293T	Human embryonic kidney cell line
HUVEC	Human umbilical vein endothelial cell
IL	Interleukin
ILV	Intraluminal vesicle
LPS	Lipopolysaccharide
M. tb	Mycobacterium tuberculosis
miR	MicroRNA
MMP	Matrix metalloproteinases
MSC	Mesenchymal stem cell
MVs	Multivesicular body
NK	Natural killer cells
nSMase	Neutral sphingomyelinase
NVEP	Non-vesicular extracellular particle
PM	Plasma membrane
TF	Tissue factor
TGF	Transforming growth factor
TNF	Tumor necrosis factor
VEGF	Vascular endothelial growth factor
